# Low-temperature features of the psychrophilic chaperonin from *Pseudoalteromonas haloplanktis*

**DOI:** 10.1007/s00203-024-04019-y

**Published:** 2024-06-11

**Authors:** Eva Hertle, Astrid Ursinus, Jörg Martin

**Affiliations:** Department of Protein Evolution, Max Planck Institute for Biology, Max-Planck-Ring 5, 72076 Tübingen, Germany

**Keywords:** Chaperonin, Psychrophilic, GroEL, ATPase, Protein folding

## Abstract

Chaperonins from psychrophilic bacteria have been shown to exist as single-ring complexes. This deviation from the standard double-ring structure has been thought to be a beneficial adaptation to the cold environment. Here we show that Cpn60 from the psychrophile *Pseudoalteromonas haloplanktis* (Ph) maintains its double-ring structure also in the cold. A strongly reduced ATPase activity keeps the chaperonin in an energy-saving dormant state, until binding of client protein activates it. Ph Cpn60 in complex with co-chaperonin Ph Cpn10 efficiently assists in protein folding up to 55 °C. Moreover, we show that recombinant expression of Ph Cpn60 can provide its host *Escherichia coli* with improved viability under low temperature growth conditions. These properties of the Ph chaperonin may make it a valuable tool in the folding and stabilization of psychrophilic proteins.

## Introduction

The Cpn60/Cpn10 chaperonin is a central component of the bacterial proteostasis network. It prevents the aggregation and supports the folding of newly synthesized polypeptides. In addition, it maintains misfolded proteins in a soluble state under stress conditions, for example heat shock (Gaitanaris et al. 1994; Kerner et al. 2005; Kim et al. 2013; Bhandari and Houry 2015). To perform this plethora of functions, high constitutive expression levels of the chaperonin genes are required, ranking chaperonins among the most abundant cellular proteins. Bacterial chaperonins consist of Cpn60, a homotetradecameric double-ring cylinder composed of 57 kDa subunits and its co-chaperonin Cpn10, a homoheptameric dome-shaped ring composed of approximately 10 kDa subunits (Braig et al. 1994; Hunt et al. 1996; Xu et al. 1997; Saibil et al. 2013). Paradigms for Cpn60 and Cpn10 are the Escherichia coli (Ec) proteins GroEL and GroES, respectively. Each Cpn60 subunit has an apical domain for client protein, facing the inside of the cylinder, and for Cpn10 binding on top. An equatorial domain harbors the ATP binding site, which forms the basis for ATPase-driven structural changes that are transmitted between the domains. Kinetic analyses have revealed nested allosteric behavior of GroEL, displaying intra-ring positive cooperativity and inter-ring negative cooperativity with respect to ATP hydrolysis (Yifrach and Horovitz 1995; Horovitz et al. 2001). Unfolded client proteins bind to hydrophobic patches at the inside of the cylinder, thereby shielding them from the exterior cytosol. In the next step of the reaction cycle, Cpn10 caps the Cpn60 cavity, effectively enclosing the client protein. Conformational changes, elicited by nucleotide binding and hydrolysis, then lead to dissociation of the client into the cavity for folding, and subsequently trigger Cpn10 release. This allows the newly folded client protein to exit the cavity and to diffuse into the cytosol. For many client proteins, a single round of folding is not sufficient to reach the native state though, resulting in rebinding of the non-native polypeptide to the chaperonin to enter once again the reaction cycle.

Over the years, competing models have been put forward to explain how chaperonins can mediate protein folding. It has been suggested that the Cpn60/Cpn10 system assists folding through a passive mechanism, where substrate encapsulation in the chaperonin cavity prevents aggregation, but does not result in changes in the folding energy landscape that would affect folding kinetics (Horwich et al. 2009; Tyagi et al. 2011). In contrast, it has been proposed that substrate encapsulation in the chaperonin cavity results in enhanced folding rates, due to confinement and the negatively charged surface of the Cpn60 cavity walls (Tang et al. 2006; Georgescauld et al. 2014; Gupta et al. 2014). Moreover, it has been proposed that chaperonins facilitate protein folding by an iterative annealing mechanism, stating that kinetically trapped folding intermediates are unfolded by the chaperonin as part of the reaction cycle (Todd et al. 1996). In addition, Cpn10 proteins can modulate Cpn60 activity and function in a rather complex manner, and not all Cpn60/Cpn10 pairs behave the same. Bacteriophage Cpn10 proteins, for example, affect their bacterial host Cpn60 differently than their cellular Cpn10 counterparts and thereby enable folding of phage proteins (Ang et al. 2000). Also, both rings of Cpn60 can become occupied simultaneously with ATP and Cpn10, thereby forming symmetric Cpn60/Cpn10_2_ particles (Yang et al. 2013). It is under debate whether these symmetric particles are functional species in the chaperonin reaction cycle (Ye and Lorimer 2013; Haldar et al. 2015). Finally, in a single-ring GroEL variant with different allosteric behavior, binding of GroES was found to actually stimulate the ATPase, allowing the progression of the reaction cycle (Kovacs et al. 2010). Thus, the various models of chaperonin action may not be necessarily mutually exclusive. Depending on the specific Cpn60/Cpn10 pair, cellular conditions, and nature of the substrate and its binding mode, more than one mechanism could be in use.

In a given organism, chaperonins have to function over a wide range of temperatures, deal with a variety of client proteins, and orchestrate binding and release of client protein, Cpn10 and nucleotides in an elaborate sequence of reaction steps. Due to these constraints, chaperonin sequences are highly conserved. On the other hand, bacteria often live in extreme environments, necessitating the chaperonins to adapt to these conditions (Kusmierczyk and Martin 2000; Ferrer et al. 2003; Strocchi et al. 2005; Sato et al. 2008; Mykytczuk et al. 2011; Luan et al. 2014). In this study, we focus on the features of psychrophilic chaperonins, exemplified by Cpn60 and Cpn10 from the gram-negative bacterium *Pseudoalteromonas haloplanktis* (Ph), an obligate marine bacterium found in Antarctic seawater (Tosco et al. 2003; Medigue et al. 2005). A thorough understanding of psychrophilic chaperonins is important insofar, as cold-adapted enzymes have been targeted for their biotechnological potentials (Cavicchioli et al. 2011). It was shown that in the cold, the *Oleispira antarctica* chaperonin Cpn60 adopts a heptameric single-ring conformation (Ferrer et al. 2004). Moreover, a more stringent ATPase regulation by the co-chaperonin Cpn10 has been proposed to serve as an energy saving mechanism under severe growth conditions (Ferrer et al. 2004; Yamauchi et al. 2012). In contrast, we show here that Ph Cpn60 is not prone to dissociating into single-rings. The Ph chaperonin has refolding activity over a wide range of temperatures, which at the upper end is limited by both Cpn10 stability and Cpn60 inactivation, and it can improve growth of *Escherichia coli* at low temperatures.

## Results and discussion

### Ph Cpn60 forms almost exclusively double-ring complexes at low temperatures

**Fig. 1:**
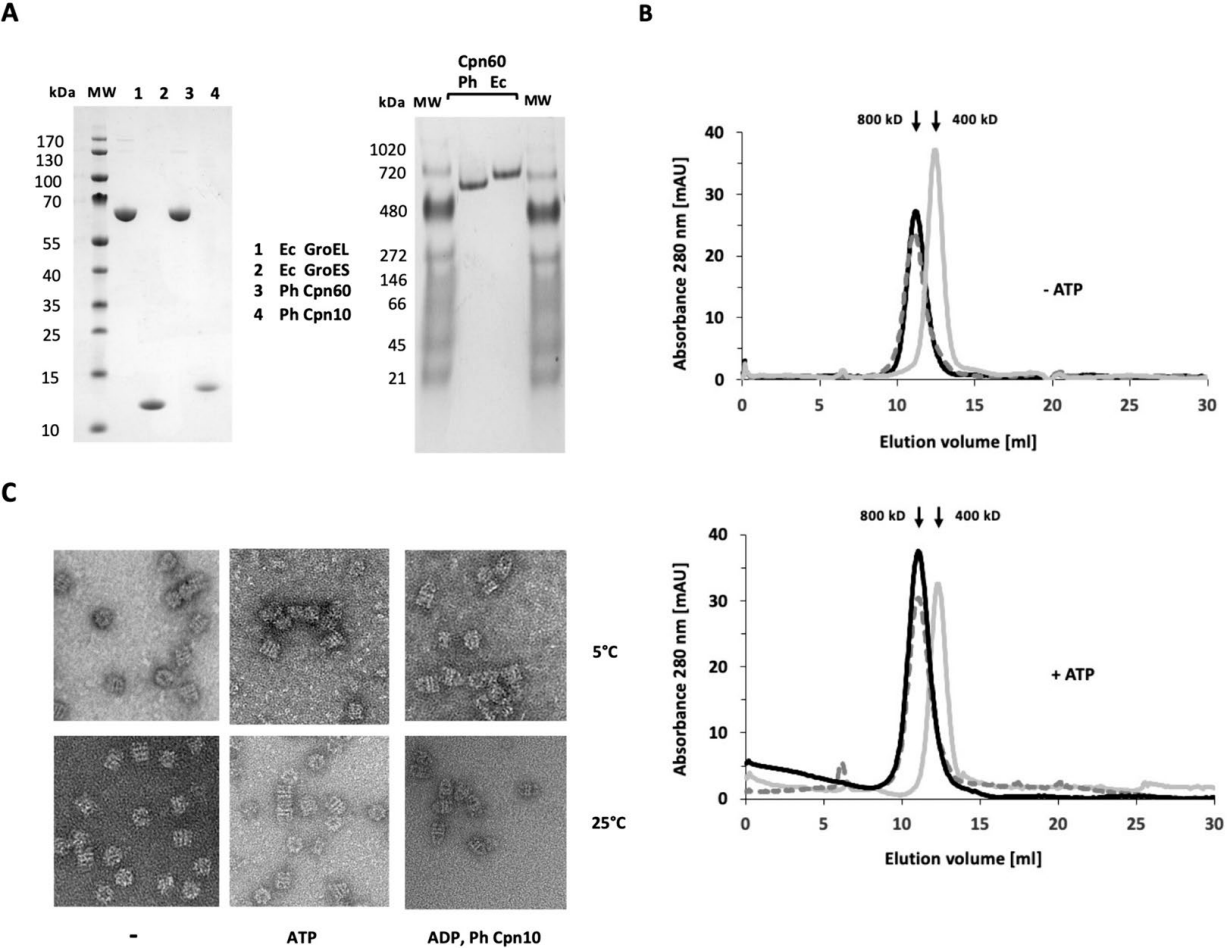
Ph Cpn60 forms almost exclusively tetradecameric complexes in the cold. **A** Purified proteins Ec GroEL and Ec GroES, and Ph Cpn60 and Ph Cpn10 were analyzed by SDS-PAGE (left panel) or native PAGE (right panel), followed by Coomassie brilliant blue staining. **B** Analytical gel filtration profile of oligomeric chaperonins in the absence (upper panel) or presence (lower panel) of 1 mM ATP. Superdex 75 columns were run at 5 °C. Dashed lines: Ph Cpn60 (MW 800 kDa); black lines: Ec GroEL (MW 800 kDa); grey lines: single-ring SR-GroEL (MW 400 kDa). **C** Electron micrographs of negatively stained Ph Cpn60 chaperonin. Upper row: incubation at 5 °C, lower row: incubation at 25 °C. Where indicated, 1 mM ATP, ADP or Ph Cpn10 were present. Side-views reveal the characteristic four-layered structure of tetradecameric Cpn60

Psychrophilic proteins are known to have a reduced stability relative to their mesophilic counterparts. In case of oligomeric complexes, changes in subunit interfaces and in oligomeric assembly have been proposed as an adaptive mechanism towards cold activity (Jaenicke and Závodszky 1990; Nowak and Otzen 2024). To probe Ph Cpn60 in this regard, we incubated the purified protein (Fig. 1A) at low temperatures and determined its oligomeric state. In analytical gel-sizing chromatography and native polyacrylamide gel electrophoresis (PAGE), Ph Cpn60 migrated at the same position as tetradecameric Ec GroEL, whereas a recombinantly generated heptameric single-ring version of Ec GroEL (SR-GroEL, MW 400 kDa) eluted on sizing columns at a later volumne, as expected (Fig. 1A, Fig. 1B, upper panel). As chaperonins undergo major conformational changes as part of the ATP hydrolysis cycle, we tested if nucleotide-dependent conformational changes could lead to ring dissociation. However, the same elution pattern was observed for Ph Cpn60 and its E. coli counterpart in the presence of Mg-ATP (Fig. 1B, lower panel), indicating that the oligomeric state of Ph Cpn60 is independent on the nucleotide state of the chaperonin. Likewise, almost exclusively double ring complexes of Ph Cpn60 were seen in negative-stain electron microscopy pictures of chaperonin that was incubated and maintained at 5 °C and 25 °C, both in the absence or presence of nucleotide, and also in complex with its co-chaperonin Ph Cpn10 (Fig. 1C). These results suggest that chaperonin equilibrium is distinctly shifted towards double-rings, in contrast to other psychrophilic chaperonins, such as the one from *Oleispira antarctica*, which at low temperatures exclusively exist as single-ring complexes (Ferrer et al. 2004), or from *Colwellia psycherythraea*, which form only moderately stable double-ring complexes that are prone to dissociation (Yamauchi et al. 2012).

## Tight regulation of Ph Cpn60 ATPase activity by Cpn10 and client protein

**Fig. 2:**
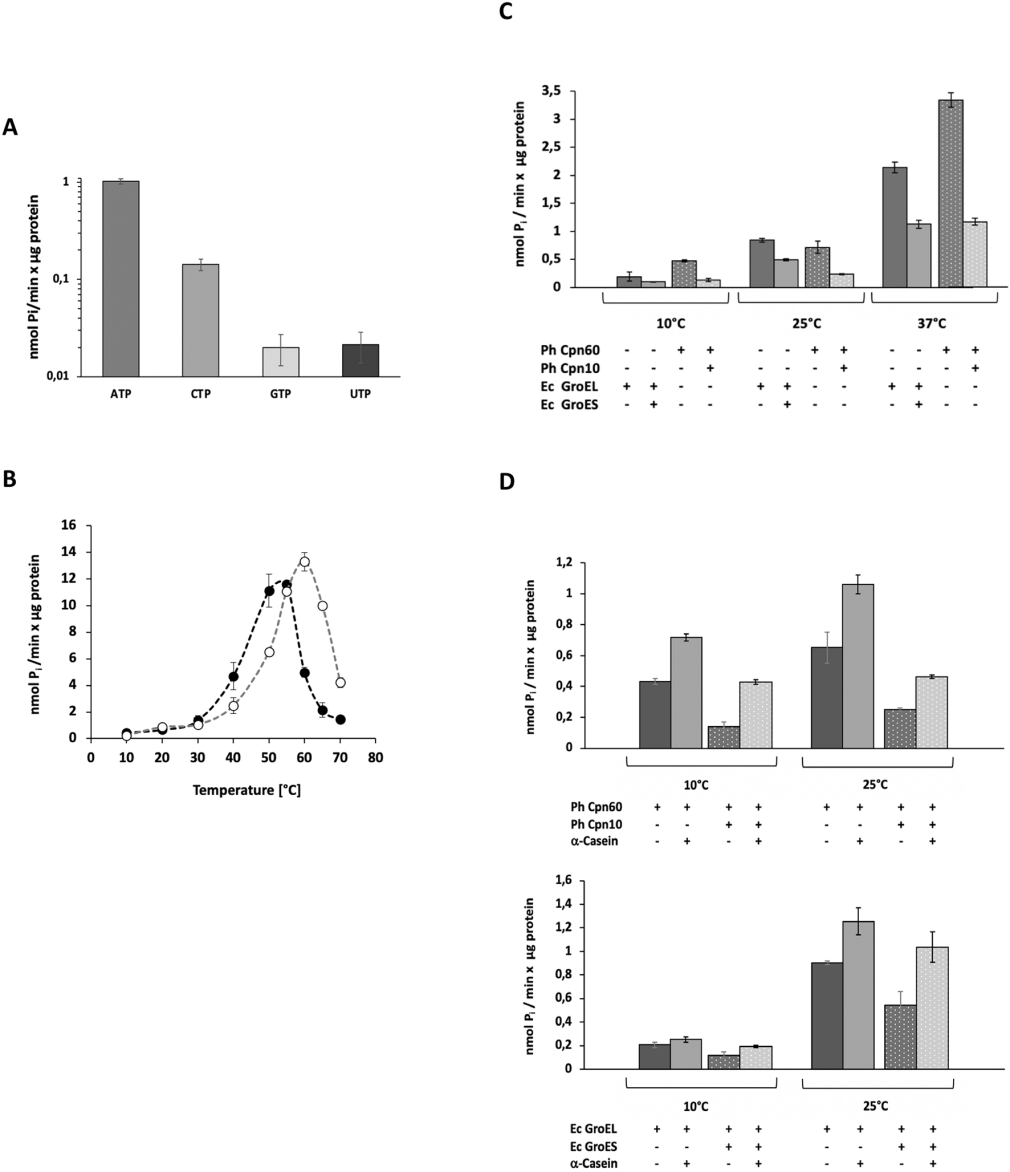
Regulation of Ph Cpn60 ATPase by Cpn10 and client protein. **A** Ability of Ph Cpn60 to hydrolyze different nucleotides ATP, CTP, GTP and UTP (1 mM each). Reactions were done at 25 °C. The released inorganic phosphate was quantified by malachite green assay. Values were determined in three independent experiments (error bars indicating SD). **B** Temperature dependence of ATPase activity of Ph Cpn60 (black circles) and Ec GroEL (open circles). Values were determined in three independent experiments (error bars indicating SD). **C** Regulation of chaperonin ATPase activity by Cpn10. Experiments were done at 10 °C, 25 °C and 37 °C in three independent experiments each (error bars indicating SD). **D** Client stimulation of Ph Cpn60 and Ec GroEL ATPase. Experiments were done in the absence or presence of a five-fold molar excess of the chaperonin client α-casein over Ph Cpn60 or Ec GroEL. Stimulation by unfolded protein was tested both in the absence or presence of Ph Cpn10 or Ec GroES. Experiments were done at 10 °C and 25 °C (error bars indicating SD)

A prerequisite for chaperoning activity and the formation of Ph Cpn60/Cpn10 complexes (Fig. 1C) is the ability of Ph Cpn60 to bind and hydrolyze ATP. Ph Cpn60 can hydrolyze ATP efficiently, with high specifity for this type of nucleotide (Fig. 2A). No activity was detected with GTP or UTP, while the CTPase activity was about 15% of the ATPase activity.

Ph Cpn60 displays ATPase activity over a wide range of temperatures (Fig. 2B) (Tosco et al. 2003). Compared to mesophilic Ec GroEL, its temperature profile is shifted towards lower temperatures though. Still, Ph Cpn60 shows maximal activity close to 55 °C, a temperature that by far exceeds the physiological temperature range experienced by *Pseudoalteromonas haloplanktis*. Beyond that temperature it rapidly loses activity.

**Fig. 3:**
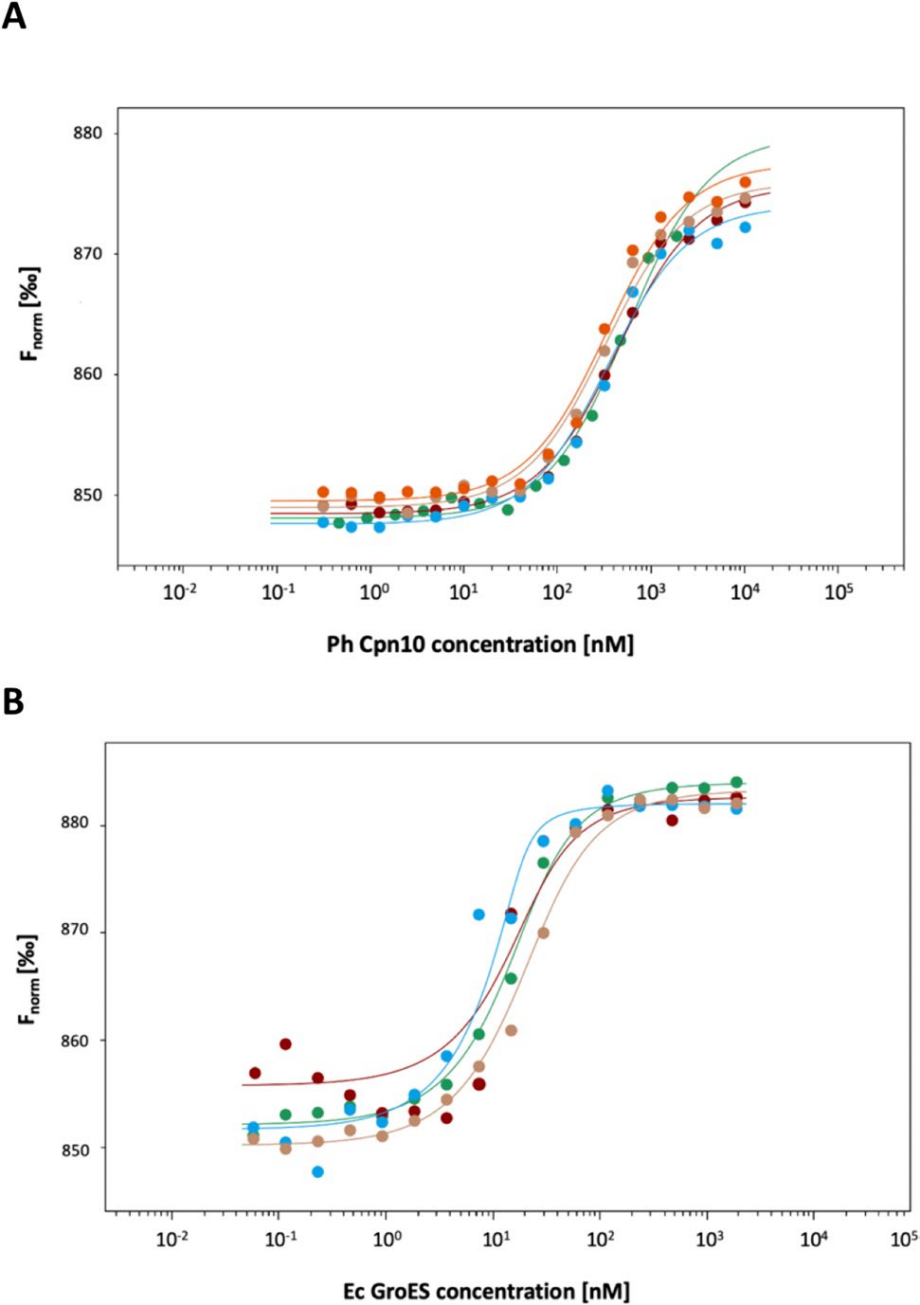
Different affinities of mesophilic and psychrophilic Cpn60 and Cpn10 complexes. The strength of interaction of Ph Cpn10 with Ph Cpn60 (**A**), or Ec GroES with Ec GroEL (**B**) was determined by Microscale Thermophoresis (MST). Increasing ligand Cpn10 concentrations were incubated with fluorescently labeled Cpn60 in the presence of ADP, resulting in a Kd of 352 ± 37 nM for the Ph chaperonin (5 measurements), and a Kd of 8.8 ± 1.8 nM for the Ec chaperonin (4 measurements). Binding curves display fluorescence changes (F_norm_) resulting from the interaction between the two proteins

In the presence of the co-chaperonin Ph Cpn10, which modulates the chaperonin reaction cycle, a strong reduction of ATP hydrolysis is seen (Fig. 2C). In agreement with findings on other psychrophilic chaperonins (Ferrer et al. 2004; Yamauchi et al. 2012), the reduction exceeds the typical 50% inhibition observed for the Ec chaperonin system. This strong inhibition has been proposed to be an ATP-saving mechanism, especially under extreme growth conditions (Ferrer et al. 2004). One possible explanation is a high affinity of the co-chaperonin Cpn10 for Ph Cpn60. However, when we quantified the interaction between Ph Cpn60 for its co-chaperonin in the presence of ADP by Microscale thermophoresis (MST), we obtained a Kd of 352 ± 37 nM (Fig. 3A). In comparison, a Kd of 8.8 ± 1.8 nM was obtained for the affinity between Ec GroEL and Ec GroES (Fig. 3B), indicating that with ADP the strength of the Ph Cpn60/Cpn10 interaction is actually weaker than that of the Ec GroEL/GroES interaction. Alternatively, the strong reduction in Ph Cpn60 ATPase activity by Ph Cpn10 could be explained by a potentially lower ATP affinity and/or hydrolysis in the trans GroEL ring of an existing Ph Cpn60/Cpn10 complex, i.e. in the ring that is not occupied by Cpn10. As ATP binding and hydrolysis in the trans Cpn60 ring are required to displace Cpn10 as part of the reaction cycle, slowing down these steps would favor a Cpn10-bound state.

Once it encounters client proteins to be folded, the chaperonin has to exit from this dormant state, as it requires ATP hydrolysis for its chaperoning activity and for the release of folding protein into its cavity. This temporary block could be overcome by conformational changes in Ph Cpn60 that are elicited by binding of an unfolded client protein. Indeed, when we tested client stimulation of the Ph Cpn60 ATPase, we found that the intrinsically disordered model client protein α-casein stimulated the ATPase of Ph Cpn60 at both 10 °C and 25 °C (Fig. 2D.). This effect was more pronounced in the cold, where we observed a three-fold increase of ATPase activity at 10 °C in the presence of Cpn10. As has been shown previously (Martin et al. 1991; Kusmierczyk and Martin 2000; Aoki et al. 2000), casein was also able to stimulate Ec GroEL ATPase, albeit to a lower extent (Fig. 2D).

## Ph chaperonin activity and protein refolding—effects of nucleotides and co-chaperonins

**Fig. 4:**
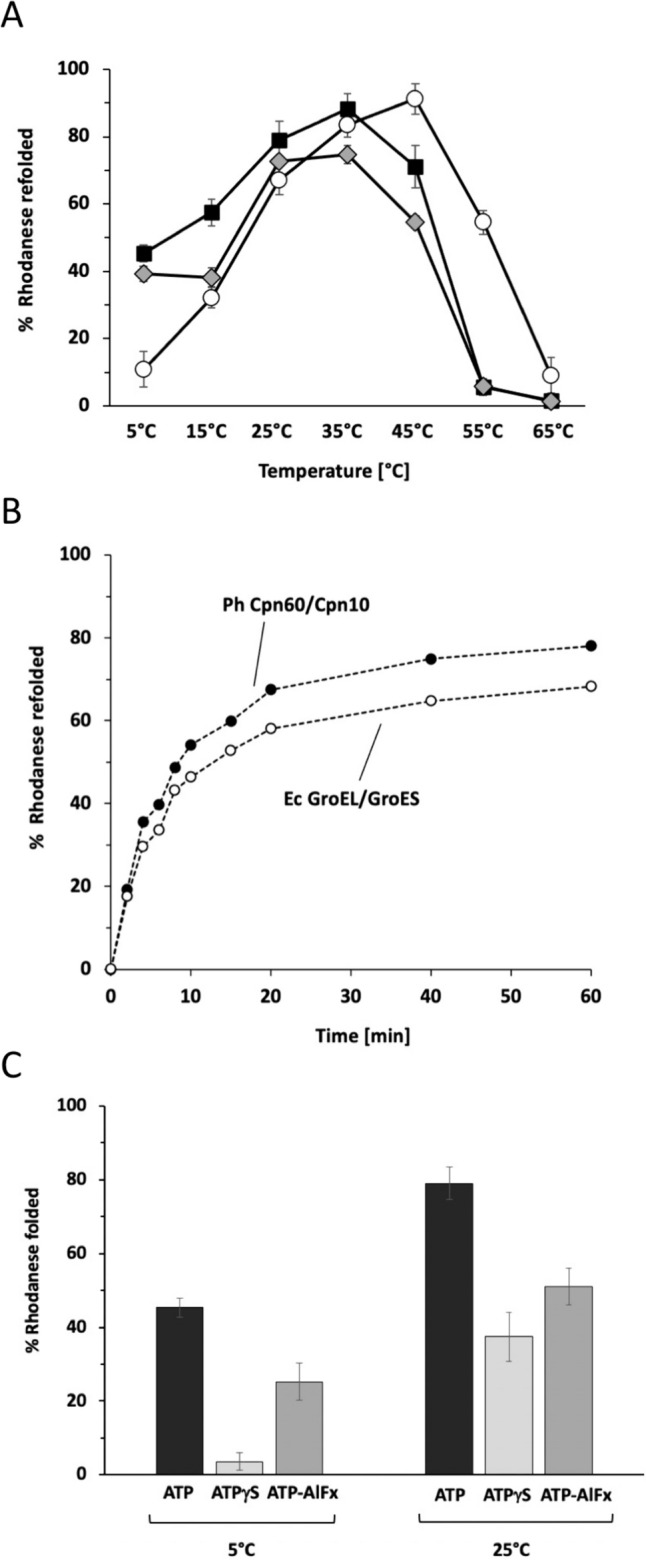
Ph chaperonin mediates protein folding over a wide range of temperatures. **A** Temperature dependence of rhodanese refolding. Chaperonin-mediated refolding was done in the presence of ATP with either Ph Cpn60 and Ph Cpn10 (black squares), Ec GroEL and Ec GroES (white circles), or Ph Cpn60 and Ec GroES (grey diamonds). Native rhodanese is set to 100%. **B** Time-course of rhodanese refolding at 25 °C with Ph Cpn60 and Ph Cpn10 (black circles), or Ec GroEL and GroES (white circles). **C** Nucleotide dependence of rhodanese refolding. Experiments were done at 5 °C or 25 °C with Ph Cpn60 and Ph Cpn10 in the presence of ATP (black), the non-hydrolyzable binding analog ATPγS (light grey), or the transition state analog ATP-AlF_x_ (dark grey)

**Fig. 5:**
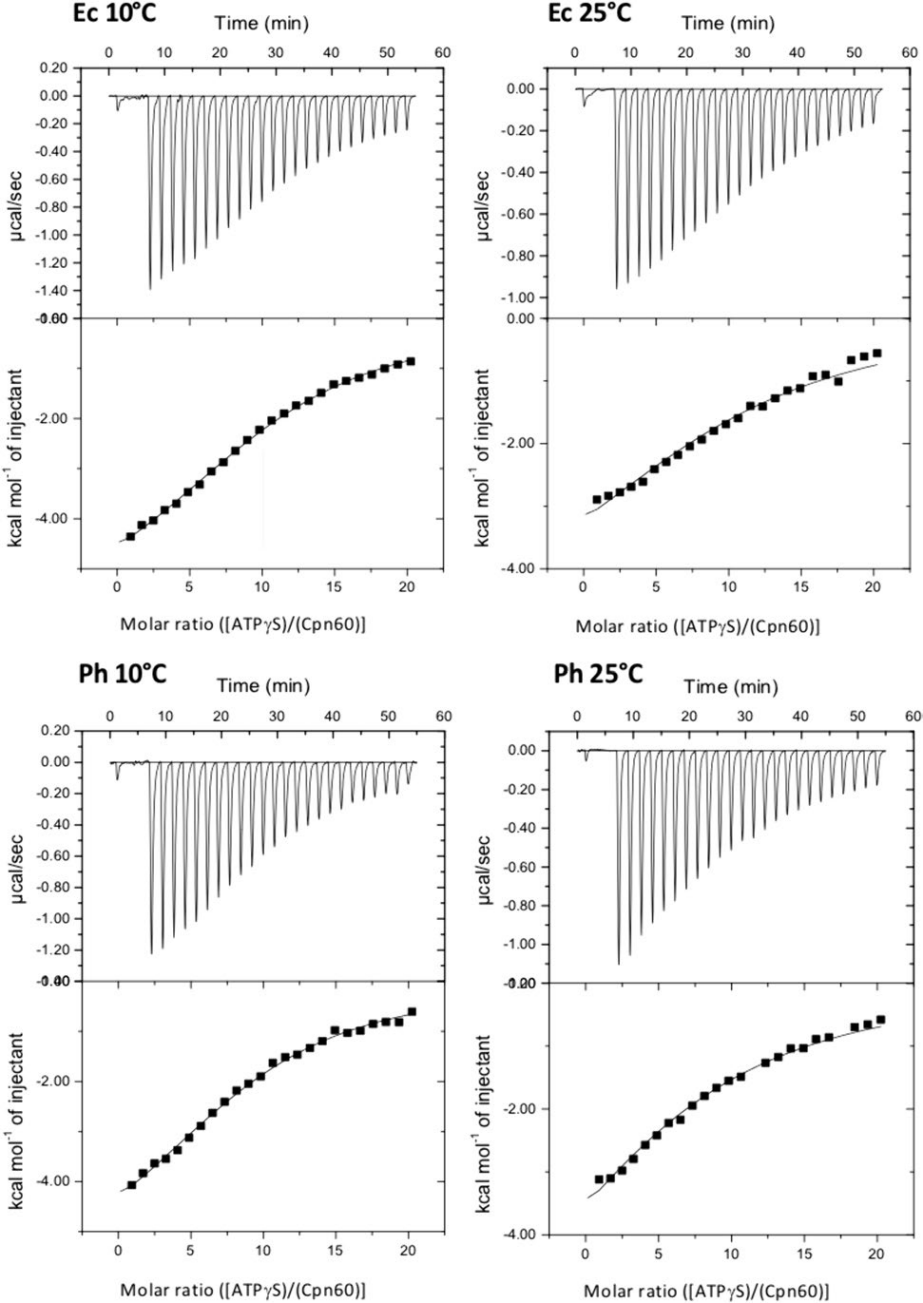
Affinity measurements of mesophilic Ec GroEL and pyschrophilic Ph Cpn60 with the non-hydrolyzable ATP binding analog ATPγS. The strength of interaction was determined by Isothermal Calorimetry (ITC). The upper parts of each panel depict profiles of the titrations at 10 °C or 25 °C, respectively, after subtraction of the control baseline; the lower parts of each panel are normalized heat changes. Data are fit to the one set of binding sites model

The ATPase temperature profile of Ph Cpn60 suggested that the chaperonin might not only be a folding assistant at low temperatures, but has the capacity to facilitate folding at higher temperatures as well. One of the best-studied Cpn60 clients is bovine rhodanese, a monomeric 33 kDa protein that cannot fold spontaneously in vitro, and that depends on the assistance of the complete Cpn60/Cpn10 chaperonin system (Martin et al. 1991; Mendoza et al. 1991; Peralta et al. 1994). When we tested the refolding of this stringent client with Ph Cpn60 and Ph Cpn10 at different temperatures, we found that the Ph chaperonin can efficiently mediate rhodanese folding not only in the cold, but also up to 50 °C (Fig. 4A). As is exemplarily shown for rhodanese refolding at 25 °C (Fig. 4B), refolding yield and kinetics are similar to those obtained with the Ec chaperonin system. We find that folding activity of the Ph chaperonin is dependent on ATP hydrolysis, either in the form of ATP or the transition state analog ATP-AlF_x_ (Fig. 4C), which was observed to stabilize the association of Ec GroES with GroEL (Chaudhry et al. 2003) and has been used to trap refolding client proteins within the chaperonin cage (Tyagi et al. 2009; Okamoto et al. 2017). While the non-hydrolyzable binding analog ATPγS can substitute for ATP as nucleotide at 25 °C to some extent, it does not mediate rhodanese refolding at 5 °C (Fig. 4C). In a regular chaperonin reaction cycle, upon ATP binding to Cpn60, followed by Cpn10 binding on top of the same cis GroEL ring, bound client protein is released into the chaperonin cavity for folding. After ATP hydrolysis in the cis ring, ATP binding in the trans Cpn60 ring triggers dissociation of Cpn10, followed by release of client protein into solution. In the presence of ATPγS, without ATP hydrolysis, a ternary Cpn60/Cpn10/rhodanese complex persists and released rhodanese continues to stay in the cavity. One reason for the inability of ATPγS to mediate folding in the cold could be a lower affinity for the analog. We measured affinities of Ph Cpn60 and Ec GroEL for ATPγS at 10 °C and 25 °C by isothermal calorimetry (ITC) and obtained at both temperatures similar Kd values in the range of 40–80 µM (Fig. 5 and Table 1), arguing against this hypothesis. ATPγS affinity is similar to that of ADP (Terada and Kuwajima 1999), and–though the type of measurements are not directly comparable–considerably weaker than the affinity of ATP for GroEL (Dyachenko et al. 2013). Other parameters of ATPγS binding might influence Ph Cpn60 chaperonin activity though, such as its non-cooperative binding mode, or the number of bound nucleotide that varies with temperature (Terada and Kuwajima 1999) and that determines the stability of the high affinity R state for ligand binding (Dyachenko et al. 2013). It is also conceivable that at lower temperatures rhodanese has to undergo several cycles of rebinding and release from the chaperonin to reach its native state, which is not possible in a persistent ternary Cpn60/Cpn10/client complex that is formed in the presence of ATPγS.

**Table 1:** Best-fit parameters obtained for ATPγS binding to Ph Cpn60 and Ec GroEL determined by Isothermal Calorimetry (ITC)

Protein	Temperature (°C)	K_d_ (M)	∆G^0^ (kcal mol^−1^)	∆H^0^ (kcal mol^−1^)	− T∆S^0^ (kcal mol^−1^)	n	
Ph Cpn60	10	3.9 × 10^–5^ ± 0.6	5.76 ± 0.06	− 6.2 ± 0.21	0.53 ± 0.02	8.94 ± 0.26	
Ph Cpn60	25	8.1 × 10^–5^ ± 0.4	− 5.62 ± 0.06	− 7.5 ± 0.25	1.88 ± 0.07	7.54 ± 0.31	
Ec GroEL	10	3.8 × 10^–5^ ± 0.2	− 5.69 ± 0.02	− 6.3 ± 0.14	0.61 ± 0.02	10.4 ± 0.15	
Ec GroEL	25	6.6 × 10^–5^ ± 0.4	− 5.61 ± 0.03	− 5.3 ± 0.18	− 0.31 ± 0.04	10.4 ± 0.44	

**Fig. 6:**
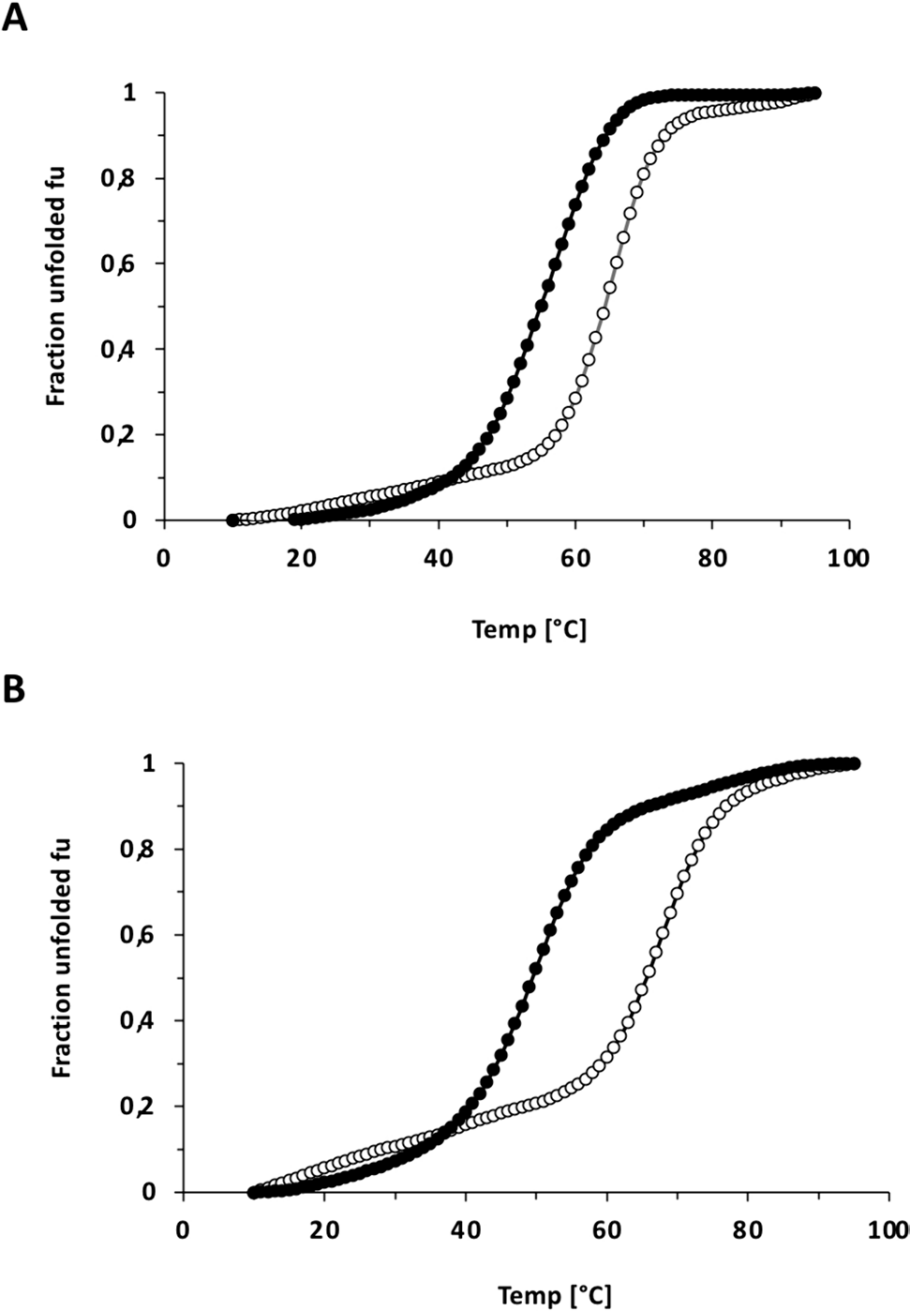
Different stabilities of mesophilic and psychrophilic Cpn60 and Cpn10 proteins. Circular dichroism melting curves of Ph Cpn60 and Ec GroEL (**A**) and Ph Cpn10 and Ec GroES proteins (**B**) were recorded from 10–95 °C at a wavelength of 222 nm. Data were converted to the fraction of unfolded protein f_U_. T_m_ midpoints are at 56 °C for Ph Cpn60, 63 °C for Ec GroEL, 49 °C for Ph Cpn10, and 61 °C for Ec GroES

Notably, for the Ec chaperonin we observed rhodanese refolding activity up to 60 °C, i.e. 10 °C higher than for the Ph chaperonin (Fig. 4A). What could cause this difference in activity between mesophilic and psychrophilic chaperonin at the upper end of the assayed temperature spectrum? To assess the stability of Ph Cpn60 and Ph Cpn10 at elevated temperatures, we measured their heat-induced denaturation using circular dichroism melting curves (Fig. 6). Cooperative unfolding was observed for all proteins. The mesophilic GroEL and GroES have similar stabilities, Tm 63 °C and 61 °C respectiviely, and Ph Cpn60 is only slightly less temperature resistant than Ec GroEL, with a Tm of 56 °C. In contrast, the stability of the co-chaperonin Ph Cpn10 significantly differs from that of its mesophilic counterpart, showing a moderately low Tm of 49 °C. This suggested that the progressing instability of Ph Cpn10 at elevated temperatures could become a limiting factor. As Ec GroES appeared to be more stable, we therefore also tested rhodanese refolding with a combination of Ph Cpn60 and Ec GroES. While both proteins can cooperate in rhodanese refolding, our results show that chaperonin activity still ceases beyond 50 °C, resembling the results obtained with Ph Cpn10 (Fig. 4A). Thus, high temperature does not only affect Ph Cpn10, but also seems to perturb Ph Cpn60 in a significant manner. Being the more complex process, involving also a refolding client protein and Cpn10, loss of chaperonin activity occurs already at lower temperatures than the loss of Ph Cpn60 ATPase activity (Fig. 2B). We also note that Ph Cpn60 is a determining factor for chaperonin activity in the cold, as Ec GroES is more effective in assisting rhodanese refolding at 5 °C when it cooperates with the psychrophilic Ph Cpn60, than with the mesophilic Ec GroEL (Fig. 4A).

## Recombinant expression of Ph GroEL can provide *Escherichia* coli with improved viability under low temperature growth conditions

**Fig. 7:**
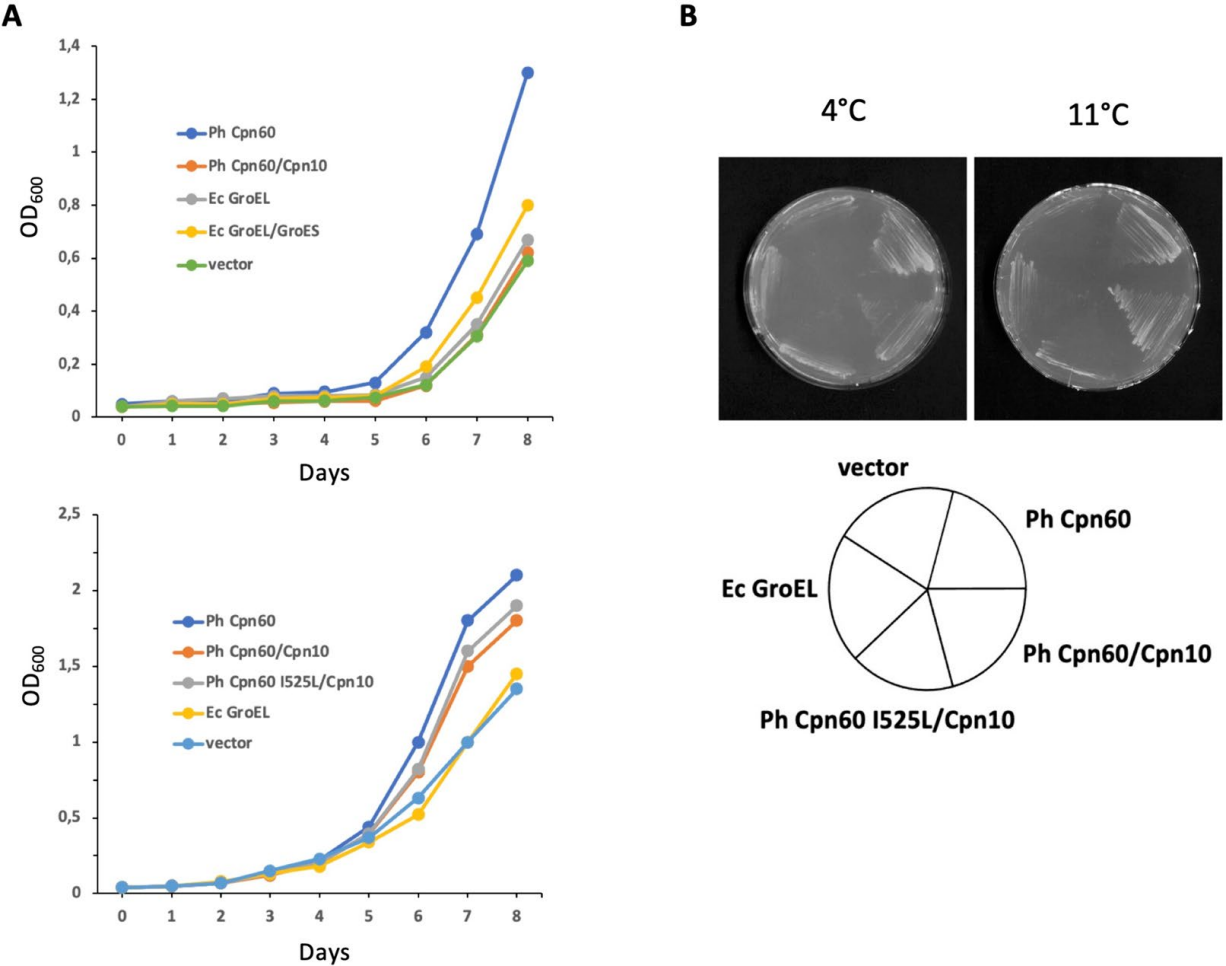
Recombinantly expressed Ph Cpn60 can provide cold resistance to *Escherichia coli*. **A** *E. coli* cells carrying plasmids expressing different chaperonin variants, as well as a control vector, were grown at 11 °C in LB medium for an extended period. To induce expression of chaperonin genes cloned in pBAD vectors, 0.2% arabinose was added, for induction of genes in pIBA vectors 0.2 µg/ml anhydrotetracycline was used. **B** Growth of *E. coli* cells expressing different chaperonin variants in pBAD vectors on LB agar plates at 4 °C and 11 °C. To induce expression of chaperonin genes, 0.2% arabinose was added. After 7 days (11 °C) and 12 days (4 °C), cell viability was visualized photographically

Given the large number of intracellular clients that depend on chaperonins for folding, a cold-adapted chaperonin should be pivotal for the sustained growth of bacteria at low temperatures (Ferrer et al. 2003; Nakamura et al. 2004). To evaluate the physiological role of Ph Cpn60 in this respect, we examined the low temperature growth of an engineered *E. coli* strain that contained variants of different chaperonin genes on a plasmid. The results in Fig. 7 indicate that expression of Ph Cpn60 indeed conferred better growth in the cold. Expression of mesophilic Ec GroEL or a Ph Cpn60 I525L mutant was somewhat less efficient. Previously, it was shown that with a I525L mutation, which is located in the C-terminal tail of Cpn60 subunits, a psychrophilic GroEL adopts features of a mesophilic chaperonin; simultaneously it loses in vitro refolding activity at 10 °C (Nakamura et al. 2004). In comparison to co-expression of Ph Cpn60/Cpn10, Ph Cpn60 was more effective when expressed on its own. This may hint at a holding function for Ph Cpn60 at lower temperatures, where non-native proteins could be prevented from aggregation and kept in solution by cycling on and off the chaperonin. A prerequisite for such a function is a productive interaction of Cpn60 with unfolded client proteins at these temperatures. The binding properties of psychrophilic Ph Cpn60 should be tailored to these conditions. Maintaining high enzymatic activity at low temperatures is typically achieved by a more flexible structure of cold-active proteins, which comes at the price of a lower stability (activity-stability trade-off) (Siddiqui and Cavicchioli 2006). Based on the results of protein engineering efforts (Wintrode and Arnold 2001; Akanuma et al. 2019) it has been suggested that neutral drift may be an important factor in the reduction of stability for psychrophilic enzymes, as they do not experience selection for stability at higher temperatures (Miyazaki et al. 2000). In general, enzymes of cold-adapted microorganisms display reduced numbers of intramolecular bonds, more loop extensions, and increased active site accessibility (Struvay and Feller 2012; Siddiqui et al. 2013; Lee et al. 2017) to aid substrate binding. Still, the binding affinity of substrates for cold-active enzymes is often lower than that of their thermophilic counterparts (Siddiqui and Cavicchioli 2006). Typically, interaction of chaperonins with their clients is mediated by the interaction of exposed hydrophobic surfaces in the unfolded client protein with hydrophobic patches at the inside of the Cpn60 cylinder. However, hydrophobic interactions are weakened when temperature is decreased, whereas electrostatic interactions are strengthened (Papaleo et al. 2007, 2011; Collins and Feller 2023). Thus, Ph Cpn60 might bind a different set of client proteins, or bind it differently, resulting also in different dynamics of release for a given protein.

In summary, we have shown that the chaperonin from the psychrophile *P. haloplanktis* is both stable and active over a wide range of temperatures. It can efficiently assist in protein folding in vitro at temperatures up to 55 °C, whereas in the cold, recombinantly expressed Ph chaperonin is beneficial for the cold resistance of its host *Escherichia coli*. With these properties, Ph chaperonin may find applications in the folding and stabilization of biotechnologically relevant cold-adapted proteins.

## Materials and methods

### Molecular biology and microbiology techniques

Genes encoding for Cpn60 and Cpn10 from *Pseudoalteromonas haloplanktis* DSM6060 were amplified by PCR using genomic DNA as a template. Constructs were cloned into pet30b expression vectors (Novagen). Likewise, Ec GroEL, single-ring SR-GroEL, and Ec GroES genes were also cloned in *E. coli* expression vectors as previously described (Weissman et al. 1995; Hayer-Hartl et al. 1996; Kusmiercyk and Martin 2000*)*. To determine chaperonin effects on the cold adaptation of *E. coli*, strain 10-beta (New England Biolabs), a DH10B derivative, was transformed with pBAD/HisA vector (Invitrogen) containing arabinose-inducible genes for either Ph Cpn60, Ph Cpn60/Cpn10, Ph Cpn60 I525L/Cpn10, Ec GroEL, or a vector control. The I525L Ph Cpn60 mutant was generated by site-directed mutagenesis (Hemsley et al. 1989). Equal amounts of cells were grown on LB agar plates supplemented with 100 mg ml^−1^ampicillin and 0.2% arabinose at 4 and 11 °C. In parallel, liquid growth curves were obtained at 11 °C, with chaperonin genes expressed in either pBAD vectors (induction with 0.2% arabinose) or in pIBA2 vectors (IBA Lifesciences) (induction with 0.2 µg/ml anhydrotetracycline).

## Protein expression and purification

Ec GroEL, single-ring SR-GroEL, and Ec GroES were expressed in *E. coli* and purified as described (Weissman et al. 1995; Hayer-Hartl et al. 1996; Kusmiercyk and Martin 2000).

For the purification of *P. haloplanktis* chaperonins Ph Cpn60 and Ph Cpn10, BL21-Gold (DE3) cells were grown in LB medium at 25 °C. Over-night expression was induced by addition of 1 mM isopropyl-thiogalactoside (IPTG) at OD600 0.6. Bacterial cell pellets were resuspended in buffer A (50 mM Tris pH 8, 50 mM NaCl, 5 mM MgCl_2_) supplemented with DNaseI (Applichem) and protease inhibitor cocktail (cOmplete, Roche). After breaking the cells in a French Press, soluble centrifugation supernatants were purified by anion-exchange (Q Sepharose HP) chromatography in buffer B (30 mM Tris pH 8) using a salt gradient up to 500 mM NaCl, followed by gel size exclusion chromatography (Superdex S300 for Ph GroEL, Superdex 75 for Ph GroES) in buffer C (20 mM MOPS pH 7.2, 150 mM KCl, 5 mM MgCl_2_). For Ph Cpn10, an additional ammonium sulfate precipitation step (35%) was used to concentrate and purify the protein after the anion-exchange column run. In a final additional step in purification for Ph Cpn60, contaminating peptides were removed on a Reactive Red 120 column (Clark et al. 1998).

Analytical Gel filtration to compare chaperonin oligomerization states was performed in buffer C on a Superdex 75 GL (10/300) column.

## Biophysical and enzyme assays

For electron microscopy (EM), glow-discharged carbon-coated grids were incubated with 0.125 µM Cpn60 in buffer C, preincubated at the given temperatures When indicated, 1 mM ATP, 1 mM ADP and 0.375 µM Cpn10 were present. Cpn60 concentrations refer to tetradecamer throughout, Cpn10 concentrations refer to heptamers throughout. Samples were negatively stained with 1% uranyl acetate and examined with a FEI Tecnai G2 Spirit BioTwin transmission EM at 120 kV. Images were collected on a Gatan Ultrascan 4000 camera.

Circular dichroism (CD) spectra were recorded with a Jasco J-810 spectropolarimeter in buffer C. Cuvettes of 1 mm path length were used in all measurements. For melting curves and determination of Tm, CD measurements were recorded at 222 nm from 10° to 95 °C, the temperature change was set to 1 °C per minute, using a Peltier-controlled sample holder unit. The fraction of unfolded protein f_U_ was determined based on f_U_ = (y_F_–y)/(y_F_–y_U_), where y_F_ and y_U_ are the values of y typical of the folded and unfolded states.

To determine the affinity and the K_d_ of the Cpn60 and Cpn10 interaction, Microscale Thermophoresis (MST) was used. For MST measurements, Cpn60 was labeled with fluorophore using the NT-647-NHS labeling kit (Nanotemper). Serial dilutions of the ligand binding partner Cpn10 were prepared in buffer C containing 1 mM ADP. MST measurements were performed at a Monolith NT.115 (Nanotemper) at a temperature of 25 °C, using MST power 80% and laser intensity 40%. Binding curves were fitted to the data, using the NT Analysis 1.5.41 software.

Affinities of mesophilic Ec GroEL and pyschrophilic Ph Cpn60 for the non-hydrolyzable ATP binding analog ATPγS were determined by Isothermal Calorimetry (ITC). Measurements were done at 10 °C and 25 °C in 20 mM MOPS pH 7.2, 100 mM NaCl, 5 mM MgCl_2_, using a Microcal VP-ITC instrument (Malvern). 9 μM Cpn60 protein was placed in the instrument’s cell, 1 mM ATPγS dissolved in the same buffer in the syringe. A first injection of 2 μl was followed by 24 10 μl injections. Stoichiometric and thermodynamic parameters of the interaction were calculated based on a one set of binding sites model using the ORIGIN software provided by the manufacturer.

ATPase activity was measured at the temperatures indicated in the figure legends by quantification of released Pi, using the malachite green reagent method (Lanzetta et al. 1979), measuring absorbance at 640 nm. Assays were done in buffer C, containing 0.125 µM Cpn60 and 1 mM ATP. To determine the nucleotide specificity of Ph Cpn60, ATP was replaced by 1 mM CTP, GTP or UTP. Whenever indicated, 0.375 µM Cpn10 or 0.625 µM α-casein (Sigma) were added to the reaction mixture.

For protein refolding experiments, client protein bovine rhodanese (Sigma) (80 µM) was unfolded in 6 M guanidinium chloride, 1 mM DTT and bound to Cpn60 by 150-fold dilution into buffer C containing 50 mM sodium thiosulfate. Samples contained in each case a three-fold molar excess Cpn60. Refolding was initiated by adding 1 mM ATP, ATPγS or ATP-fluoroaluminate (ATP-AlF_x_) and Cpn10 at a three-fold molar excess over total Cpn60. The extent of reactivation was determined by monitoring the regain of enzymatic activity at defined time points using a colorimetric assay (Martin et al. 1991; Peralta et al. 1994).

## Data Availability

No datasets were generated or analysed during the current study.

## References

[CR1] Akanuma S, Bessho M, Kimura H, Furukawa R, Yokobori SI, Yamagishi A (2019) Establishment of mesophilic-like catalytic properties in a thermophilic enzyme without affecting its thermal stability. Sci Rep 9:9346. 10.1038/s41598-019-45560-x 31249343 10.1038/s41598-019-45560-x PMC6597716

[CR2] Ang D, Keppel F, Klein G, Richardson A, Georgopoulos C (2000) Genetic analysis of bacteriophage-encoded cochaperonins. Annu Rev Genet 34:439–456. 10.1146/annurev.genet.34.1.439 11092834 10.1146/annurev.genet.34.1.439

[CR3] Aoki K, Motojima F, Taguchi H, Yomo T, Yoshida M (2000) GroEL binds artificial proteins with random sequences. J Biol Chem 275:13755–13758. 10.1074/jbc.275.18.13755 10788496 10.1074/jbc.275.18.13755

[CR4] Bhandari V, Houry WA (2015) Substrate Interaction networks of the escherichia coli chaperones: trigger Factor, DnaK and GroEL. Adv Exp Med Biol 883:271–294. 10.1007/978-3-319-23603-2_15 26621473 10.1007/978-3-319-23603-2_15

[CR5] Braig K, Otwinowski Z, Hegde R, Boisvert DC, Joachimiak A, Horwich AL, Sigler PB (1994) The crystal structure of the bacterial chaperonin GroEL at 2.8 A. Nature 371:578–586. 10.1038/371578a0 7935790 10.1038/371578a0

[CR6] Cavicchioli R, Charlton T, Ertan H, Mohd Omar S, Siddiqui KS, Williams TJ (2011) Biotechnological uses of enzymes from psychrophiles. Microb Biotechnol 4:449–460. 10.1111/j.1751-7915.2011.00258.x 21733127 10.1111/j.1751-7915.2011.00258.x PMC3815257

[CR7] Chaudhry C, Farr GW, Todd MJ, Rye HS, Brunger AT, Adams PD, Horwich AL, Sigler PB (2003) Roles of the gamma-phosphate of ATP in triggering protein folding by GroEL-GroES: function, structure and energetics. EMBO J 22:4877–4887. 10.1093/emboj/cdg477 14517228 10.1093/emboj/cdg477 PMC204461

[CR8] Clark AC, Ramanathan R, Frieden C (1998) Purification of GroEL with low fluorescence background. Methods Enzymol 290:100–118. 10.1016/s0076-6879(98)90010-6 9534154 10.1016/s0076-6879(98)90010-6

[CR9] Collins T, Feller G (2023) Psychrophilic enzymes: strategies for cold-adaptation. Essays Biochem 67:701–713. 10.1042/EBC20220193 37021674 10.1042/EBC20220193

[CR10] Dyachenko A, Gruber R, Shimon L, Horovitz A, Sharon M (2013) Allosteric mechanisms can be distinguished using structural mass spectrometry. Proc Natl Acad Sci USA 110:7235–7239. 10.1073/pnas.1302395110 23589876 10.1073/pnas.1302395110 PMC3645570

[CR11] Ferrer M, Chernikova TN, Yakimov MM, Golyshin PN, Timmis KN (2003) Chaperonins govern growth of Escherichia coli at low temperatures. Nat Biotechnol 21:1266–1267. 10.1038/nbt1103-1266 14595348 10.1038/nbt1103-1266

[CR12] Ferrer M, Lünsdorf H, Chernikova TN, Yakimov M, Timmis KN, Golyshin PN (2004) Functional consequences of single:double ring transitions in chaperonins: life in the cold. Mol Microbiol 53:167–182. 10.1111/j.1365-2958.2004.04077.x 15225312 10.1111/j.1365-2958.2004.04077.x

[CR13] Gaitanaris GA, Vysokanov A, Hung SC, Gottesman ME, Gragerov A (1994) Successive action of escherichia coli chaperones in vivo. Mol Microbiol 14:861–869. 10.1111/j.1365-2958.1994.tb01322.x 7715448 10.1111/j.1365-2958.1994.tb01322.x

[CR14] Georgescauld F, Popova K, Gupta AJ, Bracher A, Engen JR, Hayer-Hartl M, Hartl FU (2014) GroEL/ES chaperonin modulates the mechanism and accelerates the rate of TIM-barrel domain folding. Cell 157:922–934. 10.1016/j.cell.2014.03.038 24813614 10.1016/j.cell.2014.03.038 PMC4071350

[CR15] Gupta AJ, HaldarMilicǐc SG, Hartl FU, Hayer-Hartl M (2014) Active cage mechanism of chaperonin-assisted protein folding demonstrated at single-molecule level. J Mol Biol 426:2739–2754. 10.1016/j.jmb.2014.04.018 24816391 10.1016/j.jmb.2014.04.018

[CR16] Haldar S, Gupta AJ, Yan X, Milicǐc G, Hartl FU, Hayer-Hartl M (2015) Chaperonin-assisted protein folding: Relative population of asymmetric and symmetric GroEL:GroES complexes. J Mol Biol 427:2244–2255. 10.1016/j.jmb.2015.04.009 25912285 10.1016/j.jmb.2015.04.009

[CR17] Hayer-Hartl MK, Weber F, Hartl FU (1996) Mechanism of chaperonin action: GroES binding and release can drive GroEL-mediated protein folding in the absence of ATP hydrolysis. EMBO J 15:6111–6121 8947033 10.1002/j.1460-2075.1996.tb00999.x PMC452432

[CR18] Hemsley A, Arnheim N, Toney MD, Cortopassi G, Galas DJ (1989) A simple method for site-directed mutagenesis using the polymerase chain reaction. Nucleic Acids Res 17:6545–6551. 10.1093/nar/17.16.6545 2674899 10.1093/nar/17.16.6545 PMC318348

[CR19] Horovitz A, Fridmann Y, Kafri G, Yifrach O (2001) Allostery in chaperonins. J Struct Biol 135:104–114. 10.1006/jsbi.2001.4377 11580260 10.1006/jsbi.2001.4377

[CR20] Horwich AL, Apetri AC, Fenton WA (2009) The GroEL/GroES cis cavity as a passive anti-aggregation device. FEBS Lett 583:2654–2662. 10.1016/j.febslet.2009.06.049 19577567 10.1016/j.febslet.2009.06.049 PMC2759771

[CR21] Hunt JF, Weaver AJ, Landry SJ, Gierasch L, Deisenhofer J (1996) The crystal structure of the GroES co-chaperonin at 2.8 A resolution. Nature 379:37–45. 10.1038/379037a0 8538739 10.1038/379037a0

[CR22] Inbar E, Horovitz A (1997) GroES promotes the T to R transition of the GroEL ring distal to GroES in the GroEL-GroES complex. Biochem 36:12276–12281. 10.1021/bi9714870 9315866 10.1021/bi9714870

[CR23] Jaenicke R, Závodszky P (1990) Proteins under extreme physical conditions. FEBS Lett 268:344–349. 10.1016/0014-5793(90)81283-t 2200715 10.1016/0014-5793(90)81283-t

[CR24] Kerner MJ et al (2005) Proteome-wide analysis of chaperonin-dependent protein folding in escherichia coli. Cell 122:209–220. 10.1016/j.cell.2005.05.028 16051146 10.1016/j.cell.2005.05.028

[CR25] Kim YE, Hipp MS, Bracher A, Hayer-Hartl M, Hartl FU (2013) Molecular chaperone functions in protein folding and proteostasis. Annu Rev Biochem 82:323–355. 10.1146/annurev-biochem-060208-092442 23746257 10.1146/annurev-biochem-060208-092442

[CR26] Kovács E, Sun Z, Liu H, Scott DJ, Karsisiotis AI, Clarke AR, Burston SG, Lund PA (2010) Characterisation of a GroEL single-ring mutant that supports growth of escherichia coli and has GroES-dependent ATPase activity. J Mol Biol 396:1271–1283. 10.1016/j.jmb.2009.11.074 20006619 10.1016/j.jmb.2009.11.074

[CR27] Kusmierczyk AR, Martin J (2000) High salt-induced conversion of escherichia coli GroEL into a fully functional thermophilic chaperonin. J Biol Chem 275:33504–33511. 10.1074/jbc.M006256200 10945996 10.1074/jbc.M006256200

[CR28] Lanzetta PA, Alvarez LJ, Reinach PS, Candia OA (1979) An improved assay for nanomole amounts of inorganic phosphate. Anal Biochem 100:95–97. 10.1016/0003-2697(79)90115-5 161695 10.1016/0003-2697(79)90115-5

[CR29] Lee C, Jang SH, Chung HS (2017) Improving the stability of cold-adapted enzymes by immobilization. Catalysts. 10.3390/catal7040112 10.3390/catal7040112

[CR30] Luan G, Dong H, Zhang T, Lin Z, Zhang Y, Li Y, Cai Z (2014) Engineering cellular robustness of microbes by introducing the GroESL chaperonins from extremophilic bacteria. J Biotechnol 178:38–40. 10.1016/j.jbiotec.2014.03.010 24637367 10.1016/j.jbiotec.2014.03.010

[CR31] Martin J, Langer T, Boteva R, Schramel A, Horwich AL, Hartl FU (1991) Chaperonin-mediated protein folding at the surface of groEL through a ’molten globule’-like intermediate. Nature 352:36–42. 10.1038/352036a0 1676490 10.1038/352036a0

[CR32] Médigue C et al (2005) Coping with cold: the genome of the versatile marine Antarctica bacterium Pseudoalteromonas haloplanktis TAC125. Genome Res 15:1325–1335. 10.1101/gr.4126905 16169927 10.1101/gr.4126905 PMC1240074

[CR33] Mendoza JA, Rogers E, Lorimer GH, Horowitz PM (1991) Chaperonins facilitate the in vitro folding of monomeric mitochondrial rhodanese. J Biol Chem 266:13044–13049 1677004 10.1016/S0021-9258(18)98800-9

[CR34] Miyazaki K, Wintrode PL, Grayling RA, Rubingh DN, Arnold FH (2000) Directed evolution study of temperature adaptation in a psychrophilic enzyme. J Mol Biol 297:1015–1026. 10.1006/jmbi.2000.3612 10736234 10.1006/jmbi.2000.3612

[CR35] Mykytczuk NC, Trevors JT, Foote SJ, Leduc LG, Ferroni GD, Twine SM (2011) Proteomic insights into cold adaptation of psychrotrophic and mesophilic Acidithiobacillus ferrooxidans strains. Antonie Van Leeuwenhoek 100:259–277. 10.1007/s10482-011-9584-z 21604047 10.1007/s10482-011-9584-z

[CR36] Nakamura T, Tanaka M, Maruyama A, Higashi Y, Kurusu Y (2004) A nonconserved carboxy-terminal segment of GroEL contributes to reaction temperature. Biosci Biotechnol Biochem 68:2498–2504. 10.1271/bbb.68.2498 15618620 10.1271/bbb.68.2498

[CR37] Nowak S, Otzen DE (2024) Helping proteins come in from the cold: 5 burning questions about cold-active enzymes. BBA Advances 5:100104. 10.1016/j.bbadva.2023.100104 38162634 10.1016/j.bbadva.2023.100104 PMC10755280

[CR38] Okamoto T, Yamamoto H, Kudo I, Matsumoto K, Odaka M, Grave E, Itoh H (2017) HSP60 possesses a GTPase activity and mediates protein folding with HSP10. Sci Rep 7:16931. 10.1038/s41598-017-17167-7 29208924 10.1038/s41598-017-17167-7 PMC5717063

[CR39] Papaleo E, Olufsen M, De Gioia L, Brandsdal BO (2007) Optimization of electrostatics as a strategy for cold-adaptation: a case study of cold- and warm-active elastases. J Mol Graph Model 26:93–103. 10.1016/j.jmgm.2006.09.012 17084098 10.1016/j.jmgm.2006.09.012

[CR40] Papaleo E, Tiberti M, Invernizzi G, Pasi M, Ranzani V (2011) Molecular determinants of enzyme cold adaptation: comparative structural and computational studies of cold- and warm-adapted enzymes. Curr Protein Pept Sci 12:657–683. 10.2174/1389203711109070657 21827423 10.2174/1389203711109070657

[CR41] Peralta D, Hartman DJ, Hoogenraad NJ, Høj P (1994) Generation of a stable folding intermediate which can be rescued by the chaperonins GroEL and GroES. FEBS Lett 339:45–49. 10.1016/0014-5793(94)80381-1 7906229 10.1016/0014-5793(94)80381-1

[CR42] Saibil HR, Fenton WA, Clare DK, Horwich AL (2013) Structure and allostery of the chaperonin GroEL. J Mol Biol 425:1476–1487. 10.1016/j.jmb.2012.11.028 23183375 10.1016/j.jmb.2012.11.028

[CR43] Sato S, Ikeuchi M, Nakamoto H (2008) Expression and function of a groEL paralog in the thermophilic cyanobacterium Thermosynechococcus elongatus under heat and cold stress. FEBS Lett 582:3389–3395. 10.1016/j.febslet.2008.08.034 18786533 10.1016/j.febslet.2008.08.034

[CR44] Siddiqui KS, Cavicchioli R (2006) Cold-adapted enzymes. Annu Rev Biochem 75:403–433. 10.1146/annurev.biochem.75.103004.142723 16756497 10.1146/annurev.biochem.75.103004.142723

[CR45] Siddiqui KS et al (2013) Psychrophiles. Annu Rev Earth Planet Sci 41:87–115. 10.1146/annurev-earth-040610-133514 10.1146/annurev-earth-040610-133514

[CR46] Strocchi M, Ferrer M, Timmis KN, Golyshin PN (2006) Low temperature-induced systems failure in Escherichia coli: insights from rescue by cold-adapted chaperones. Proteomics 6:193–206. 10.1002/pmic.200500031 16302275 10.1002/pmic.200500031

[CR47] Struvay C, Feller G (2012) Optimization to low temperature activity in psychrophilic enzymes. Int J Mol Sci 13:11643–11665. 10.3390/ijms130911643 23109875 10.3390/ijms130911643 PMC3472767

[CR48] Tang YC, Chang HC, Roeben A, Wischnewski D, Wischnewski N, Kerner MJ, Hartl FU, Hayer-Hartl M (2006) Structural features of the GroEL-GroES nano-cage required for rapid folding of encapsulated protein. Cell 125:903–914. 10.1016/j.cell.2006.04.027 16751100 10.1016/j.cell.2006.04.027

[CR49] Terada TP, Kuwajima K (1999) Thermodynamics of nucleotide binding to the chaperonin GroEL studied by isothermal titration calorimetry: evidence for noncooperative nucleotide binding. Biochim Biophys Acta 1431:269–281. 10.1016/s0167-4838(99)00049-7 10350604 10.1016/s0167-4838(99)00049-7

[CR50] Todd MJ, Lorimer GH, Thirumalai D (1996) Chaperonin- facilitated protein folding: optimization of rate and yield by an iterative annealing mechanism. Proc Natl Acad Sci USA 93:4030–4035. 10.1073/pnas.93.9.4030 8633011 10.1073/pnas.93.9.4030 PMC39481

[CR51] Tosco A, Birolo L, Madonna S, Lolli G, Sannia G, Marino G (2003) GroEL from the psychrophilic bacterium Pseudoalteromonas haloplanktis TAC 125: molecular characterization and gene cloning. Extremophiles 7:17–28. 10.1007/s00792-002-0291-6 12579376 10.1007/s00792-002-0291-6

[CR52] Tyagi NK, Fenton WA, Horwich AL (2009) GroEL/GroES cycling: ATP binds to an open ring before substrate protein binding and production of the native state. Proc Natl Acad Sci USA 106:20264–20269. 10.1073/pnas.0911556106 19915138 10.1073/pnas.0911556106 PMC2777187

[CR53] Tyagi NK, Fenton WA, Deniz AA, Horwich AL (2011) Double mutant MBP refolds at same rate in free solution as inside the GroEL/GroES chaperonin chamber when aggregation in free solution is prevented. FEBS Lett 585:1969–1672 21609718 10.1016/j.febslet.2011.05.031 PMC3144026

[CR54] Weissman JS et al (1995) Mechanism of GroEL action: productive release of polypeptide from a sequestered position under GroES. Cell 83:577–587 7585961 10.1016/0092-8674(95)90098-5

[CR55] Wintrode PL, Arnold PH (2001) Temperature adaptation of enzymes: lessons from laboratory evolution. Adv Protein Chem 55:161–225. 10.1016/s0065-3233(01)55004-4 10.1016/s0065-3233(01)55004-4 11050934

[CR56] Xu Z, Horwich AL, Sigler PB (1997) The crystal structure of the asymmetric GroEL-GroES-(ADP)7 chaperonin complex. Nature 388:741–750. 10.1038/41944 9285585 10.1038/41944

[CR57] Yamauchi S, Ueda Y, Matsumoto M, Inoue U, Hayashi H (2012) Distinct features of protein folding by the GroEL system from a psychrophilic bacterium, Colwellia psychrerythraea 34H. Extremophiles 16:871–882. 10.1007/s00792-012-0483-7 22996829 10.1007/s00792-012-0483-7

[CR58] Yang D, Ye X, Lorimer GH (2013) Symmetric GroEL:GroES_2_ complexes are the protein-folding functional form of the chaperonin nanomachine. Proc Natl Acad Sci USA 110:E4298–E4305. 10.1073/pnas.1318862110 24167279 10.1073/pnas.1318862110 PMC3832010

[CR59] Ye X, Lorimer GH (2013) Substrate protein switches GroE chaperonins from asymmetric to symmetric cycling by catalyzing nucleotide exchange. Proc Natl Acad Sci USA 110:E4289–E4297. 10.1073/pnas.1317702110 24167257 10.1073/pnas.1317702110 PMC3831975

[CR60] Yifrach O, Horovitz A (1995) Nested cooperativity in the ATPase activity of the oligomeric chaperonin GroEL. Biochem 34:5303–5308. 10.1021/bi00016a001 7727391 10.1021/bi00016a001

